# Correlation of End-Tidal Carbon Dioxide with Arterial Carbon Dioxide in Mechanically Ventilated Patients

**DOI:** 10.5812/atr.6444

**Published:** 2012-08-21

**Authors:** Ebrahim Razi, Gholam Abbass Moosavi, Keivan Omidi, Ashkan Khakpour Saebi, Armin Razi

**Affiliations:** 1 Trauma Research Center, Kashan University of Medical Sciences, Kashan, IR Iran; 2 Faculty of Medicine, Shahid Beheshti University of Medical Sciences, Tehran, IR Iran

**Keywords:** Blood Gas Analysis, Carbon Dioxide, Artificial Respiration

## Abstract

**Background:**

Patients undergone mechanical ventilation need rapid and reliable evaluation of their respiratory status. Monitoring of End-tidal carbon dioxide (ETCO_2_) as a surrogate, noninvasive measurement of arterial carbon dioxide (PaCO_2_) is one of the methods used for this purpose in intubated patients.

**Objectives:**

The aim of the present trial was to study the relationship between end-tidal CO_2_ tensions with PaCO_2_ measurements in mechanically ventilated patients.

**Materials and Methods:**

End-tidal carbon dioxide levels were recorded at the time of arterial blood gas sampling. Patients who were undergoing one of the mechanical ventilation methods such as: synchronized mandatory mechanical ventilation (SIMV), continuous positive airway pressure (CPAP) and T-Tube were enrolled in this study. The difference between ETCO_2_ and PaCO_2_ was tested with a paired t-test. The correlation of end-tidal carbon dioxide to (ETCO_2_) CO_2_ was obtained in all patients.

**Results:**

A total of 219 arterial blood gases were obtained from 87 patients (mean age, 71.7 ± 15.1 years). Statistical analysis demonstrated a good correlation between the mean of ETCO_2_ and PaCO_2_ in each of the modes of SIMV, CPAP and T-Tube; SIMV (42.5 ± 17.3 and 45.8 ± 17.1; r = 0.893, P < 0.0001), CPAP (37 ± 9.7 and 39.4 ± 10.1; r = 0.841, P < 0.0001) and T-Tube (36.1 ± 9.9 and 39.4 ± 11; r = 0.923, P < 0.0001), respectively.

**Conclusions:**

End-tidal CO_2_ measurement provides an accurate estimation of PaCO_2_ in mechanically ventilated patients. Its use may reduce the need for invasive monitoring and/or repeated arterial blood gas analyses.

## 1. Background

End-tidal CO_2_ monitors are used to estimate arterial CO_2_ pressure (PaCO_2_), but appropriate use of this noninvasive method of assessing blood gases in ventilated patients remains unclear. It has been used extensively in operating rooms, intensive care units, emergency departments and in pre-hospital setting (1-4). In a study that was conducted by Flanagan et al., end-tidal CO_2_ measurement provided an accurate estimation of PaCO_2_, even during episodes of severe hypocarbia (5). One study indicated that measurements of end-tidal carbon dioxide concentrations correlated well with PaCO_2_ values in non-intubated patients presenting with a variety of conditions to emergency departments (6). End-tidal carbon dioxide measurements may be sufficient measures of PaCO_2_ in selected patients and obviate the need for repeat arterial blood gas determination. In a study that was carried out in ventilated head trauma patients, end-tidal PaCO_2_ monitoring correlated well with PaCO_2_ in patients without respiratory complications or without spontaneous breathing (7). However, its clinical validity is questionable in patients who have the greatest need for end-tidal PaCO_2_ monitoring (i.e., patients who have respiratory distress or who are breathing spontaneously and overriding the ventilator). Noninvasive end-tidal carbon dioxide pressure (ETCO_2_) monitoring may adequately predict PaCO_2_ in non-intubated emergency department patients with respiratory distress, who are able to produce a forced expiration (8). ETCO_2_ is a less accurate measure of PaCO_2_ with tidal volume breathing and in patients with pulmonary disease. PaCO_2_ cannot be estimated by the ETCO_2_ method in a pre-hospital setting (9). There is wide variation in the gradient between PaCO_2_ and ETCO_2_ depending on the patient’s condition, and this relationship does not remain constant over time, thus it is not useful in pre-hospital ventilation management. These data do not support routine monitoring of end-tidal CO_2_ during short transportation times in adult patients requiring mechanical ventilation. However, the monitor may prevent morbidity in patients requiring tight control of PaCO_2_ (10). One study reported that, PaCO_2_ gives a poor estimate of PaCO_2_ in patients with respiratory failure (11). In another study, that was carried out in mechanically ventilated patients with multisystem trauma, trends in the arterial to end-tidal carbon dioxide gradient magnitude were not reliable, and concordant direction changes in ETCO_2_ and PaCO_2_ are not assured (12).

## 2. Objectives

The aim of the present trial was to study the relationship between end-tidal CO_2_ tensions with PaCO_2_ measurements in mechanically ventilated patients.

## 3. Materials and Methods

This was a cross-sectional study conducted on 219 arterial blood gases in 87 adult patients with respiratory failure admitted to the Intensive Care Unit (ICU) of Shahid Beheshti Hospital, Kashan University of Medical Sciences, Iran, between March 2008 and February 2010. The mean age of the patients was 71.7 ± 15.1 years, 46 of the patients were male, and 43 were female. The study was approved by the local hospital Ethics Committee and informed consent was obtained. Blood samples were drawn by radial arterial puncture. Samples were immediately analyzed for PaCO_2_ using a blood gas analyzer (AVL-995, AVL Medical Instruments, Graz, Austria). The arterial to end-tidal CO_2_ gradient was determined. The ETCO_2_ was measured using an end-tidal CO_2_ analyzer (CAPNOGARD, Respironics, California, Inc. Carisbad, CA, USA), on the expiratory side of the circuit’s endotracheal tube connector. After proper calibration and an equilibration time of 20 minutes with stable hemodynamic and respiratory variables, ETCO_2_ were determined and the highest reading was recorded. Patients who were undergoing one of the mechanical ventilation methods such as; synchronized intermittent mandatory ventilation (SIMV), continuous positive airway pressure (CPAP) and T-Tube were enrolled in this study. The Mean ± SD of PaCO_2_, ETCO_2_ values and PaCO_2_ - ETCO_2_ gradients in all of the three groups were determined. The correlation between PaCO_2_ and ETCO_2_ in one of three modes of ventilation, SIMV, CPAP or T-tube, was done using linear regression. Paired t-test and a Kruskal-Wallis test were used to compare the gradients between PaCO_2_, and ETCO_2_ in each of the SIMV, CPAP and T-tube conditions. Data are presented as Mean ± SD. P < 0.05 was considered to indicate a significant difference.

## 4. Results

**Figure 1.**
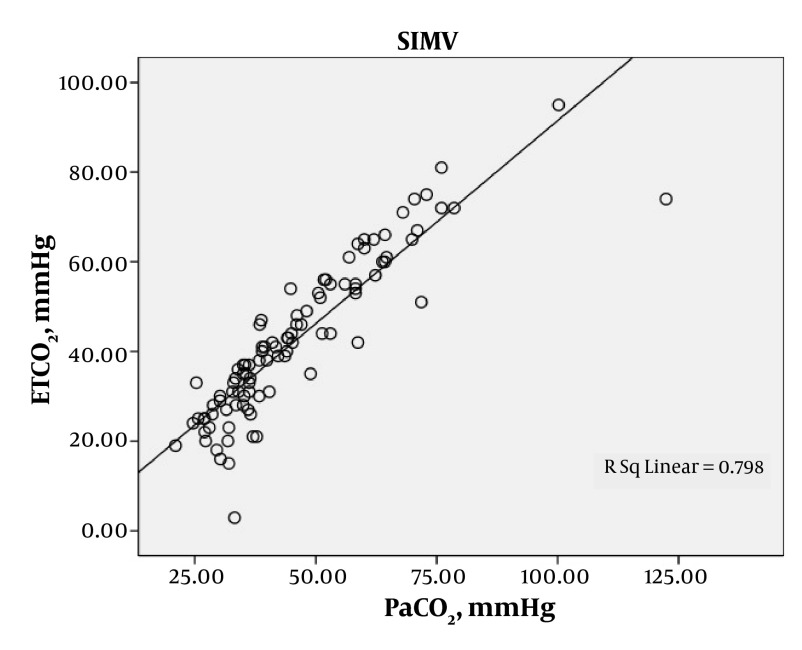
Relationship between PaCO_2_ (mmHg) and ETCO_2_ (mmHg) in SIMV Mechanically Ventilated Patients, Showing a Significant Correlation (r = 0.893, P < 0.0001)

**Figure 2.**
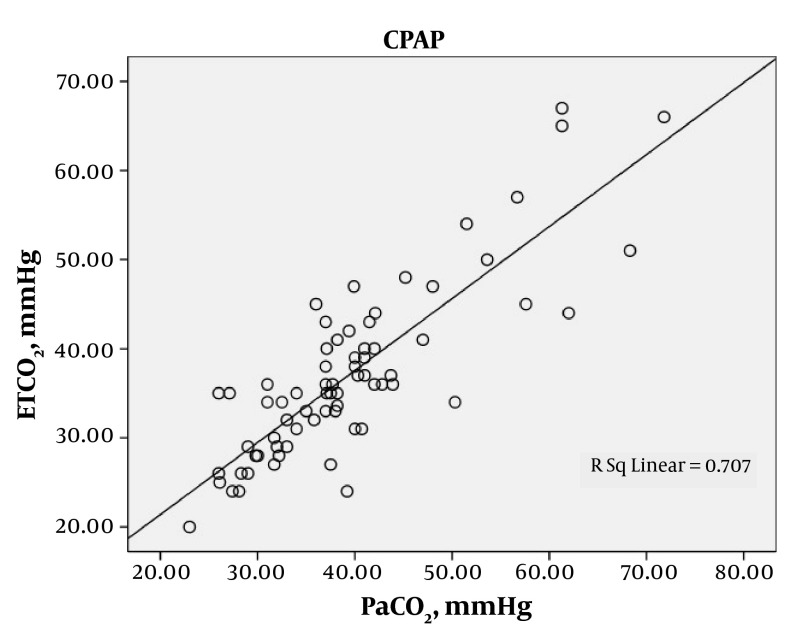
Relationship between PaCO_2_ (mmHg) and ETCO_2_ (mmHg) in CPAP Mechanically Ventilated Patients, Showing a Significant Correlation (r = 0.841, P < 0.0001)

**Figure 3.**
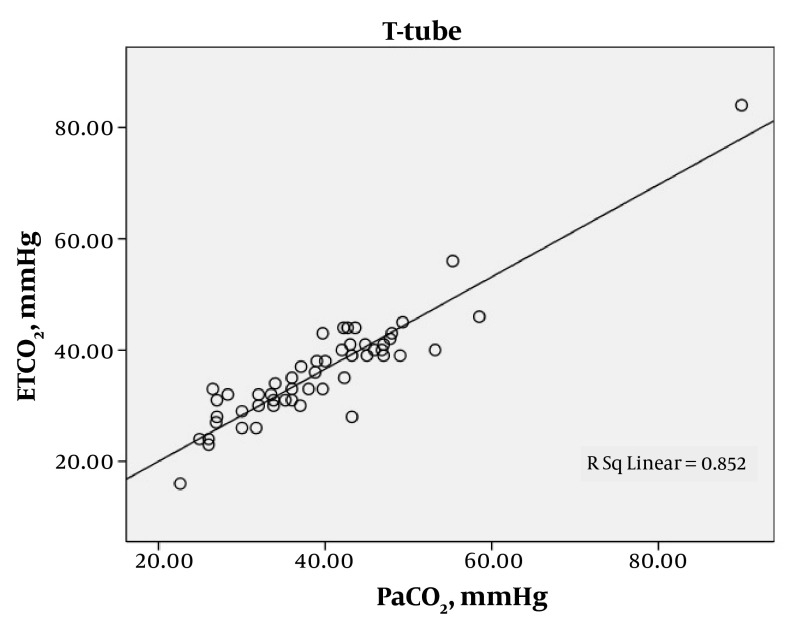
Relationship between PaCO_2_ (mmHg) and ETCO_2_ (mmHg) in TTube Ventilated Patients, Showing a Significant Correlation (r = 0.923, P < 0.0001)

A total of 219 arterial blood gases were obtained from the 87 patients. The patients were ventilated with SIMV for 97 (44.3%) gas measurements, CPAP with support pressure for 70 (32%), and T-tube for 52 (23.7%). Statistical analysis demonstrated a good correlation between the mean of ETCO_2_ and PaCO_2_ in each of the modes of SIMV, CPAP and T-Tube; SIMV (42.5 ± 17.3 and 45.8 ± 17.1; r = 0.893, P < 0.0001), CPAP (37 ± 9.7 and 39.4 ± 10.1; r = 0.841, P < 0.0001) and T-Tube (36.1 ± 9.9 and 39.4 ± 11; r = 0.923, P < 0.0001), respectively (Table 1). In each of these modes the ETCO_2_ was generally lower than the PaCO_2_. The mean difference between the arterial to end-tidal carbon dioxide tension gradients were measured in each of the modes, SIMV, CPAP and T-Tube (Table 2). A positive correlation between PaCO_2_ and ETCO_2_ was found with each of the SIMV, CPAP and T-tube modes, indicating statistical significance (Figure 1, Figure 2 and Figure 3).

**Table 1.** Comparison of PaCO_2_ with ETCO_2_ in Patients with Various Modes of Ventilation a

	PaCO_2_ a, Mean ± SD	ETCO_2_ a, Mean ± SD	R	R^2^	*P* value
**Ventilation Setting**					< 0.0001
SIMV a (n = 97)	45.8 ± 17.1	42.5 ± 17.3	0.893	0.798	
CPAP a (n = 70)	39.4 ± 10.1	37 ± 9.7	0.841	0.707	
T-tube a (n = 52)	39.4 ± 11	36.1 ± 9.9	0.923	0.852	

^a^Abbreviations: PaCO_2_, partial arterial carbon dioxide tension; ETCO_2_, end-tidal carbon dioxide; SIMV, synchronized mandatory mechanical ventilation; CPAP, continuous positive airway pressure

**Table 2.** Comparison of Mean Difference between PaCO_2_ and ETCO_2_ in Three Different Modes of Ventilation a

	PaCO_2_ a - ETCO_2_ a, Mean ± SD	95% CI
SIMV a (n = 97)	3.37 ± 7.93	1.77 - 4.97
CPAP a (n = 70)	2.32 ± 5.62	0.98 - 3.67
T-tube a (n = 52)	3.31 ± 4.26	2.13 - 4.50

^a^Abbreviations: PaCO_2_, partial arterial carbon dioxide tension; ETCO_2_, end-tidal carbon dioxide; SIMV, synchronized mandatory mechanical ventilation; CPAP, continuous positive airway pressure

The relationship between PaCO_2_ (mmHg) and ETCO_2_ (mmHg) in CPAP mechanically ventilated patients is shown in Figure 2. There is a significant correlation (r = 0.841, P < 0.0001). The relationship between PaCO_2_ (mmHg) and ETCO_2_ (mmHg) in T-Tube ventilated patients is shown in Figure 3. There is a significant correlation (r = 0.923, P < 0.0001).

## 5. Discussion

Our results clearly show a strong correlation between arterial PaCO_2_ and ETCO_2_ in critically ill patients undergoing mechanical ventilation, and it provides a clinically reliable estimate of ventilation levels. Measurements of ETCO_2_ is an accepted standard of care for the monitoring of mechanically ventilated patients and this is often used during the ventilation of critically ill patients with respiratory failure (13). In healthy subjects there are close correlation between PaCO_2_ and ETCO_2_, and it is commonly accepted that PaCO_2_ measurements vary approximately 2-5 mmHg above ETCO_2_ values (14). Generally, PaCO_2_ is expected to exceed ETCO_2_ levels. Some studies have reported the correlation between ETCO_2_ and PaCO_2_ among ventilated patients and critical states (7, 13). Generally, ETCO_2_ measurements are affected by PaCO_2_ levels, dead space fraction, and pulmonary perfusion. ETCO_2_ is dependent on alveolar CO_2_ (PACO_2_) and the site of sampling. Non-uniform alveoli CO_2_ emptying patterns, in patients with large ventilation perfusion result in mismatching PACO_2_, and underestimation of PaCO_2_ levels (13). A high ventilation/perfusion ratio and dead space tends to cause low ETCO_2_ levels relative to PaCO_2_, whereas a low ventilation/perfusion ratio and shunt has little effect on causing a smaller ETCO_2_ measure relative to PaCO_2_. Among critically ill patients, increased intrapulmonary shunting from pulmonary parenchymal disease is relatively common. Yamanaka *et al.*, reported that an admixture of this blood into the arterial circulation contributes to increased ETCO_2_ – PaCO_2_ gradients (11). This increase may be up to 20 mmHg in patients with severe pulmonary or major systemic disease. In other words, ETCO_2_ monitoring tends to underestimate PaCO_2_ levels. In a study that was conducted by Sivan *et al.*, these discrepancies started to occur below a PaO_2_/PAO_2_ ratio of 0.3 (15). Although ETCO_2_ measurements have been identified as an invaluable tool to monitor airway patency and confirm endotracheal intubation, recent studies have suggested that it may also be used to guide ventilation as a surrogate measure of PaCO_2_ (16). Previous studies have shown conflicting results concerning the correlation between PaCO_2_ and ETCO_2_ in different clinical settings. McDonald *et al.*, concluded that ETCO_2_ correlates with PaCO_2_ in critically ill patients undergoing conventional ventilation via an endotracheal tube and provides a clinically reliable estimate of ventilation (r^2^ = 0.716 and P < 0.001) (17). In their study ETCO_2_ (39.9 ± 12.7 mmHg) was lower than the PaCO_2_ (45.5 ± 14.1 mmHg). In the current study the values of ETCO_2_ and PaCO_2_ overall in the patients were 39.2 ± 13.9 mmHg versus 42.2 ± 14.1 mmHg. Kerr *et al.*, reported that ETCO_2_ and PaCO_2_ correlated well (r^2^ = 0.77) in adults with traumatic brain injury, who did not have lung disease (defined as positive end-expiratory pressure of < 5 cm/H_2_O), however, they did not correlate when a lung injury was present (7). Good correlation was also observed in a study that included adults with and without lung disease (18). Barton *et al.*, also reported that measurements of end-tidal carbon dioxide concentrations correlate well with PaCO_2_ values in non-intubated patients presenting with a variety of conditions to the emergency room (6). End-tidal carbon dioxide measurements may be sufficient measures of PaCO_2_ in selected patients and obviate the need for repeated arterial blood gas determination (6). Tobias and Meyer, concluded that measurement of transcutaneous CO_2_ was a more accurate predictor of PaCO_2_ than ETCO_2_ (19). The difference between transcutaneous CO_2_ and PaCO_2_ was less than the difference between ETCO_2_ and PaCO_2_, 2.3 ± 1.3 mmHg versus 6.8 ± 5.1 mmHg (19). Continuous monitoring of SpO_2_ (saturation of peripheral oxygen) and ETCO_2_ can be used to wean patients safely and effectively after coronary artery bypass grafting. ETCO_2_ was a good indicator of PaCO_2_ (r = 0.76), its sensitivity to detect hypercarbia (PaCO_2_ less than 45 mmHg) was 95% (20). In a study that was conducted on anesthetized, mechanically ventilated patients, transcutaneous monitoring of CO_2_ partial pressure gave a more accurate estimation of PaCO_2_ than ETCO_2_ monitoring (r^2^ = 0.73, and 0.50 respectively) (21). Weinger and Brimm measured arterial to end-tidal carbon dioxide gradient values of 4.24 ± 4.42 mmHg during intermittent mandatory ventilation (22). They reported good correlation between ETCO_2_ and PaCO_2_ in 25 adults with and without pulmonary disease (22). In patients without lung disease, ventilated either mechanically or spontaneously via a tracheal tube, the arterial to end-tidal carbon dioxide gradient values were 0.8-3.5 mmHg (23, 24). In the present study the difference between PaCO_2_ and ETCO_2_ in each of the SIMV, CPAP, and T-tube modes of the ventilator were; 3.37 ± 7.93 mmHg, 2.32 ± 5.62 mmHg, and 3.31 ± 4.26 mmHg, respectively. The objective of our study was to show that monitoring of ETCO_2_ provides a clinically useful and effective method for assessing ventilation. Several studies have indicated a poor correlation of ETCO_2_ with PaCO_2_ in an emergency setting (9, 25, 26). Russell and Graybeal, reported that in mechanically ventilated neurointensive care patients, there is significant variability in the relationship between PaCO_2_ and ETCO_2_ (27). The ETCO_2_ – PaCO_2_ gradients were reported as 6.9 ± 4.4 mmHg. In another study that was conducted by Russsell *et al.*, during intraoperative craniotomies, this value was reported to be 7.2 ± 3.3 mmHg (28). They concluded that ETCO_2_ may not provide a statistically stable estimation of PaCO_2_ in mechanically ventilated neurosurgical patients undergoing craniotomies (28). Patients with respiratory failure and multisystem trauma have a much wider difference of 14 ± 11 mmHg (12). In a recent report, Warner *et al.*, evaluated 180 intubated patients with isolated traumatic brain injury and demonstrated a poor correlation between ETCO_2_ and PaCO_2_ (r^2^ = 0.277) (29). In another study, in spite of a good correlation between ETCO_2_ and PaCO_2_ (r = 0.78, P < 0.001) in 20 intubated patients with respiratory failure, the changes in delta ETCO_2_ did not correlate as well with delta PaCO_2_ (r = 0.58, P ≤ 0.001) (30). The results of another study indicated that hypercapnia may be underestimated when ETCO_2_ is substituted for PaCO_2_ in patients breathing spontaneously via a cuffed oropharyngeal airway (31). Belpomme *et al.*, concluded that in a pre-hospital setting, PaCO_2_ cannot be estimated by ETCO_2_ levels (9). Moreover, there is a wide variation in the gradient between PaCO_2_ and ETCO_2_ depending on the patient’s condition, and over time, this relationship does not remain constant and thus it is not useful in pre-hospital ventilation management (9). In a study that was conducted on hyperventilated neurosurgical patients, the values of ETCO_2_ showed a moderately acceptable correlation with PaCO_2_ measurements. However, changes in end-tidal carbon dioxide values failed to correlate with simultaneous changes in arterial carbon dioxide tension measures (32). Palmon *et al.*, compared two groups of patients as no-monitor and monitor-blind groups that were under controlled with a capnograph during transport (10). The results of their study do not support routine monitoring of end-tidal CO_2_ during short transport times in adult patients requiring mechanical ventilation. However, the monitor may prevent morbidity in patients requiring tight control of PaCO_2_ (10). Kavanagh *et al.*, found poor correlation between end-tidal and arterial PaCO_2_, as well as poor correlation between transcutaneous and arterial PaCO_2_ in extubated, spontaneously breathing patients recovering from general anesthesia (33). In conclusion, end-tidal CO_2_ measurement provides an accurate estimation of PaCO_2_ in mechanically ventilated patients. ETCO_2_ monitoring in adult ventilated patients may be a useful tool in their management. Its use may limit the need for invasive monitoring and/or repeated arterial blood gas analyses. The results of this study shows that ETCO_2_ monitoring accurately reflects PaCO_2_ during mechanical ventilation. A comparison of mean differences between PaCO_2_ and ETCO_2_ in three different modes of ventilation did not show any statistical significance. Additional studies in relation to the efficiency of CO_2_ monitoring during various phases of mechanical ventilation are recommended.
